# Does thyroglossal duct arborization play a role in the post-surgical outcome of Sistrunk procedure in children?

**DOI:** 10.1007/s00405-024-08631-y

**Published:** 2024-04-09

**Authors:** Claudio Spinelli, Marco Ghionzoli, Clara Ugolini, Chiara Oreglio, Carla Guglielmo, Antonino Morabito, Armando Patrizio, Poupak Fallahi, Silvia Martina Ferrari, Alessandro Antonelli

**Affiliations:** 1 Division of Pediatric and Adolescent Surgery, Department of Surgery, University of Pisa, Pisa, Italy; 2 Department of Surgical, Medical, Molecular Pathology and Critical Area, University of Pisa, Pisa, Italia; 3 Department of Pediatric Surgery, Meyer Children’s Hospital, University of Florence, Florence, Italy; 4 Department of Emergency Medicine, Azienda Ospedaliero-Universitaria Pisana, Pisa, Italy; 5 Department of Translational Research and New Technologies in Medicine and Surgery, University of Pisa, Pisa, Italy; 6 Department of Clinical and Experimental Medicine, University of Pisa, Pisa, Italy

**Keywords:** Thyroglossal duct cyst, Arborization, Sistrunk’s procedure, Pediatric age

## Abstract

**Purpose:**

The purpose of the present study is to analyze thyroglossal duct cyst (TGDC) histopathological features, with focus on “arborization”, in a cohort of pediatric patients who underwent surgical removal, and evaluate a possible correlation with clinical recurrences.

**Methods:**

A retrospective analysis of all patients who underwent surgical resection for TGDC at the division of Pediatric Surgery of the University of Pisa from 2015 to 2020 was performed; for each patient, the following data were recorded: age, sex, clinical presentation, localization, size of the lesion, diagnostic tools, histopathological features, perioperative complications, recurrence and follow-up.

**Results:**

With respect to arborization, following histopathological analysis 25/30 patients (83.3%) presented thyroglossal duct branching. After a median follow-up of 3.5 years, only 2 out of 30 patients (6.7%), one male and one female, respectively aged 4 y.o. and 6 y.o., presented recurrence within one year from first surgery.

**Conclusion:**

Surgery for TGDC remains a challenge for pediatric surgeons, while arborization was present in most of our cases which underwent surgery. With respect to the role of arborization, our study did not highlight sufficient conclusive data regarding their role in recurrence: instead, it showed wide resection as satisfactory, being the arborization present in most of the cases at histopathology.

## Introduction

Thyroglossal duct cyst (TGDC) occurs in ~ 7% of the population and it represents the most common congenital anomaly of the neck in childhood, accounting for over 16% of anterior neck masses [1]. Indeed, even though it may also occur in adults, its peak of incidence occurs around 5 years of age [2].

No gender difference has been reported [3], except for Narayana et al. [4] who found a slight prevalence in male children.

During embryogenesis, the thyroid descends early in fetal life from the base of the tongue to its physiological position in the midline of the neck through a structure called thyroglossal duct (TGD).

TGDC arises from either persistence of the thyroglossal duct (TGD) or failure of its obliteration.

The epithelium lining the TGD normally disappears around the 10th gestational week and epithelial cells are reabsorbed. If there are some remnants along the pathway of the TGD opening directly at the base of the tongue through the foramen caecum, TGDC may arise. The most common sites of TGD persistence are within the thyroid (66.7%), supra-hyoid (26.1%), supra-sternal (5.3%) and intralingual (1.9%) [5]. However, TGD remnants appear to be necessary but not sufficient to cause TGDCs. Indeed, a multifactorial etiology it’s the most convincing pathogenetic hypothesis, with external stimuli inducing embryonal cells differentiation into mucous-secreting cells obstructing the TGD [6–8].

TGDCs clinical presentation is usually sufficient to obtain the diagnosis. It presents as painless, round, cystic bulging in the anterior portion of the neck. A pathognomonic sign is the movement of the mass along the neck midline following deglutition. The most common complaint is recurrent infections, causing abscesses and requiring prolonged antibiotic treatment [9].

Originally, TGDC were managed though simple cyst exeresis, but this procedure carried an unacceptably high recurrence risk of over 50%. Therefore, new techniques were proposed, first by Schlange [10] and later by Sistrunk [11, 12]. Eventually, the latter promoted the en bloc removal of a portion of the hyoid bone, together with the whole TGD from the base of the tongue to the to the cyst, and the surrounding mucosal tissue, minimizing recurrence rates [13].

There are known risk factors of recurrence, among which the inflammatory status of the cyst at the time of surgery. Moreover, several studies have suggested that the TGD may arborize above and below the hyoid bone; this might lead to persistence of some branches of the duct and subsequent recurrence after surgery, if resection performed is insufficient [14–16]

The purpose of this study is to analyze TGDCs histopathological features, with focus on “arborization”, in a cohort of pediatric patients who underwent surgical removal, and evaluate a possible correlation with clinical recurrences.

## Materials and methods

A retrospective analysis of all patients who underwent surgical resection for TGDC at the division of Pediatric Surgery of the University of Pisa from 2015 to 2020 was performed; written informed consent was obtained from the patients or from their relatives, according to the ethical committee guidelines.

**Fig. 1:**
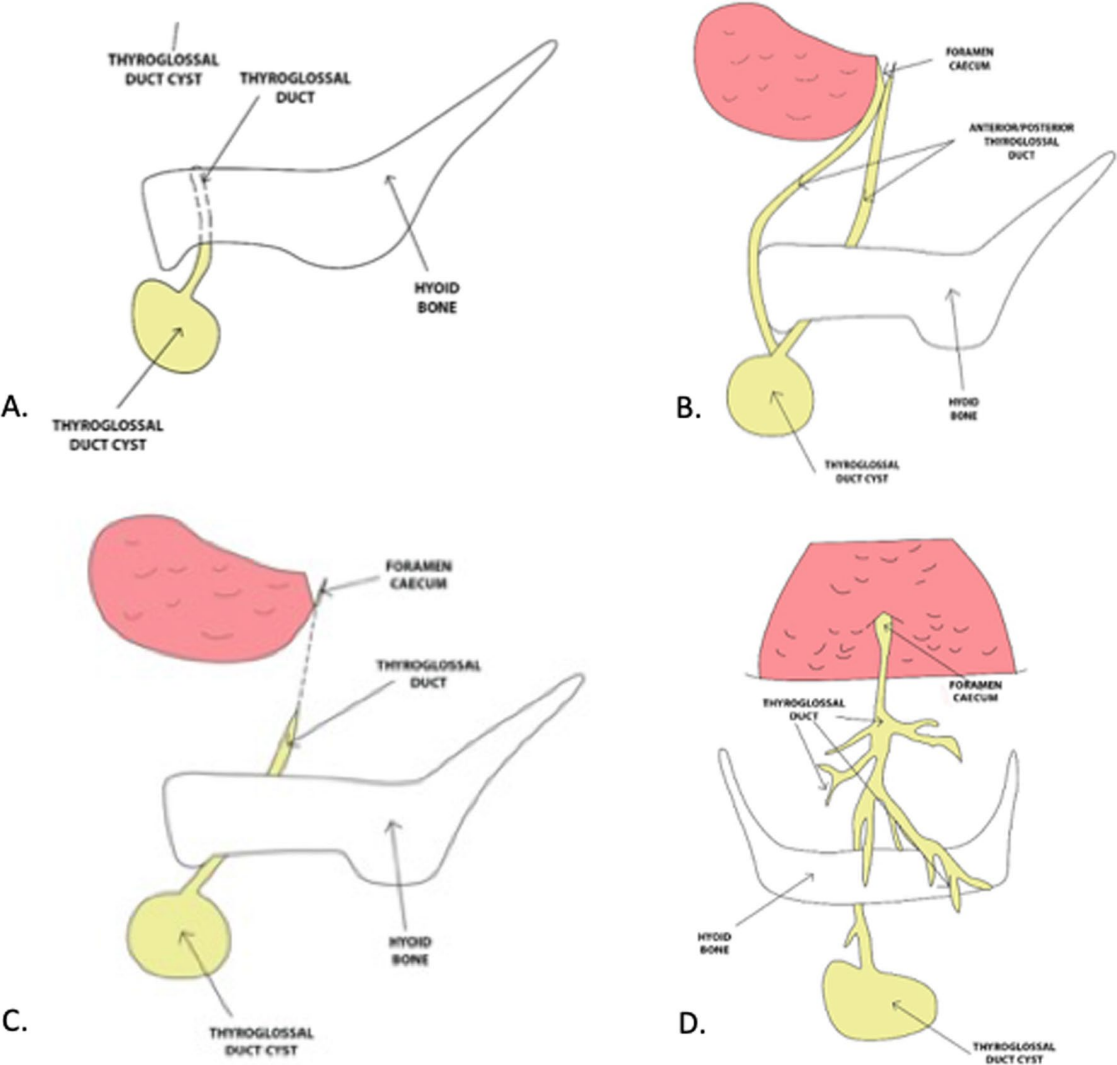
**A** Transhyoid Cyst and Thyroglossal duct. **B** Thyroglossal duct anterior and posterior to hyoid bone up to foramen caecum. **C** Thyroglossal duct with suprahyoid tract. **D** Arborization of thyroglossal duct in its pre-hyoid and retro-hyoid portion 230 × 133 mm (300 × 300 DPI)

For each patient, the following data were recorded: age, sex, clinical presentation, localization (according to Allard’s study [17]), size of the lesion, diagnostic tools, histopathological features, perioperative complications, recurrence and follow-up (Fig. 1).

All patients underwent the Sistrunk procedure: exeresis of the cyst with removal of the central portion of the hyoid bone and, when visible, the TGD remnant, up to the retrohyoid portion. The partial hyoidectomy is routinely performed via electrocautery by deconnecting the lateral parts (cartilage) from the central portion which is bony [7]. All surgical specimens underwent histopathological evaluation by a dedicated pathologist, with particular attention for signs of arborization.

Recurrence was defined as either the occurrence of a new cystic cervical lesion after surgery or a draining fistula, that required surgical excision.

A median follow-up of 3.5 years (ranging from 2 to 5 years) was performed to evaluate any possible late surgical complication or recurrence.

A review of literature was performed using PubMed database with the following keywords: “Sistrunk procedure”, “pediatric age”, “Thyroglossal Duct Cyst”, “recurrence” and “arborization of thyroglossal duct”. Inclusions criteria were: papers published from 1999 to 2021, papers presenting > 20 cases, patients < 18 y.o. The data collected were then compared to ours. Categorical data were described by absolute and relative (%) frequency. To compare the arborization (no, yes) with recurrence (no, yes) Fisher exact test was applied. Significance was fixed at 0.05 and all analyzes were carried out by SPSS v.28 technology.

## Results

The present study includes thirty patients who underwent surgery for TGDC: median age at diagnosis was 5.1 years (range: 2 months–18 years), with a peak of incidence at the age of 3. Nineteen patients (63.3%) were males, whereas eleven patients (36.7%) were females (M:F = 1.6:1). At diagnosis, twenty-three patients (76.7%) presented a painless midline cervical mass, whereas seven patients (23.3%) had severe inflammatory status prior to surgery whereas no patient underwent surgery during an acute inflammatory event. No cases presented signs of malignant transformation.

TGDC were distributed in the following locations: 21 infrahyoid (70%), six suprahyoid (20%) and 3 suprasternal (10%). Diagnosis was performed in all cases through cervical ultrasound: all patients presented a cystic mass, with a median size of 2,6 cm (ranging from 1.2 to 3.7 cm). Thirteen lesions were heterogeneous (43.3%), seven lesions were anechoic (23.3%), five were pseudo-solid mass (16.7%) and five were hypoechoic (16.7%) at US examination. Only in three patients (10%) US was inconclusive, therefore diagnostic confirmation occurred through T2-weighted MRI.

All surgical specimens were sent for histopathological analysis by a dedicated pathologist. In sixteen patients (53.3%) the histopathological analysis was unable to accurately identify TGD position with respect to the hyoid bone, due to extensive inflammation resulting from previous inflammatory bursts. In the remaining patients (46.7%) the TGD was localized anteriorly to the central part of the hyoid bone in ten cases (71.4%) and posteriorly in four patients (28.6%).

In 28 patients (93.3%) the post-surgical outcome was uneventful. Two patients (6.7%) presented infection of the surgical wound within a month from surgery, resolved with antibiotics therapy. Both patients had a history of recurrent bouts of TGDC inflammation which were also subsequently observed at histopathological review.

**Fig. 2:**
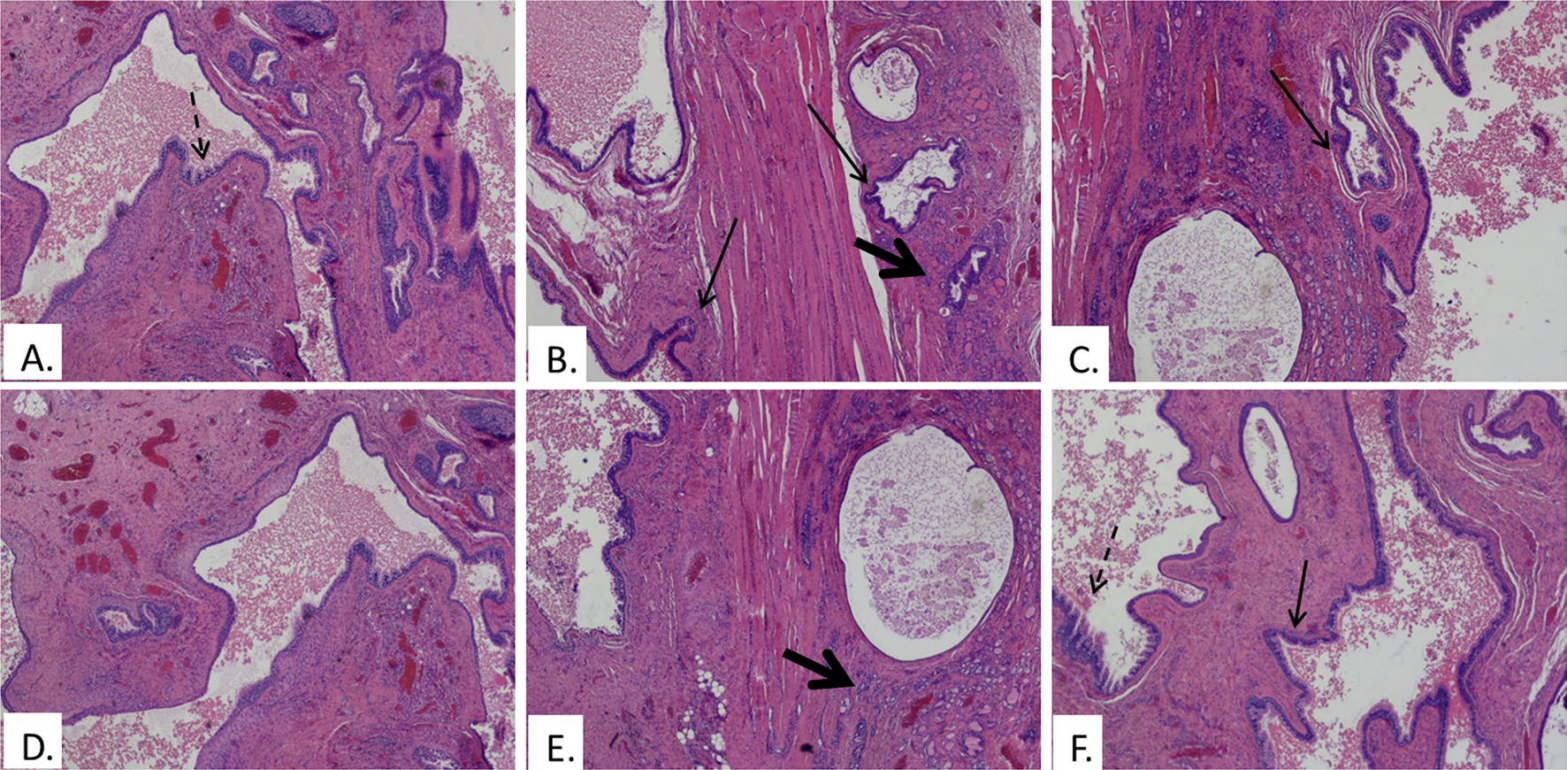
**a**–**f** Different images from a thyroglossal duct cyst. The cyst is lined by a ciliated respiratory epithelium and shows an extensive branching of the wall (black arrows) and multiple invaginations (dotted arrows). The supporting wall of the cyst contains some inflammatory cells and thyroid follicles (bold arrows). (4 × magnification; hematoxylin–eosin staining 257 × 127 mm (300 × 300 DPI)

With respect to arborization, following histopathological analysis, twenty-five patients (83.3%) presented thyroglossal duct branching (Fig. 2). After a median follow-up of 3.5 years, only 2 out of 30 patients (6.7%), one male and one female, respectively aged 4 y.o. and 6 y.o., presented recurrence within 1 year from first surgery. They both presented TGD arborization and the duct was located anteriorly to the hyoid bone with ectopic thyroid follicles. A history of recurrent inflammatory events, with scars of inflammation described at the histopathological analysis was present as well. For those cases, a second surgery was scheduled following an adequate neck MRI study and to date they are disease-free at follow-up.

To date, the remaining 23 patients (92%) with TGD arborization had no recurrence of the disease (p = 0.999).

## Discussion

Originally, TGDCs were removed through simple cyst exeresis, but presented recurrence rates as high as 50%. In 1893, Schlange [10] proposed the excision of the central portion of the hyoid bone together with the cyst and the whole duct, that reduced the rate of recurrence to nearly 20%. Eventually, in 1920 Sistrunk [11] described what became the standard surgical management of this congenital abnormality: an en bloc exeresis of the cyst, central hyoidectomy, and tract excision, extended through the tongue base tissue 2–3 mm up to the foramen cecum, including mucosa. The recurrence rate after the original Sistrunk procedure was around 1–5%; therefore in 1928, Sistrunk [12] himself modified his original technique to include the lingual muscle up to (but not including) the tongue mucosa. The modified Sistrunk procedure remains the gold standard treatment for symptomatic TGDC [18].

Incidence of re-operation after thyroglossal tract surgery has been impressively reduced since Schlange and Sistrunk [15]. However, even after a properly performed cyst exeresis through modified Sistrunk procedure, recurrence rates of TGDC reported in literature are still 7.6% (0–28%). [19–34]

It seems to be no correlation between patient’s sex or positioning of drainage, and recurrence of the disease.

Amongst recognized risk factors for clinical recurrence, instead there are: presence of extensive inflammation at time of surgery, multiple number of cysts and/or TGDs, severe inflammation at time of surgery or presence of TGDC fistula.

Firstly, inflammation at the time of surgery markedly increased the risk of recurrence. It is likely that repeated episodes of inflammation may promote scarring and obscure tissue planes, making eventually surgical excision more difficult and increasing the risk of recurrence.

This evidence may suggest the possible detrimental impact of a history of infection. In any case, the role of recurrent infections appears to be controversial, and evidence of its effects is not clear: while some reports documented a significant increased risk of recurrence after multiple infections [20, 30], others reported a slight increased risk [22, 40] and only one failed to observe any association [23]. In addition, multicystic presentation of the lesion may play a role and can be associated with a markedly increased risk of recurrence, speculating that incomplete removal of the cyst at surgery may occur especially when lesions are multicystic while children with multicystic lesions may be at increased risk of aberrant locations that are not detected at the time of first surgery [13]. Finally, risk factors analysis identified a possible role of younger age and post-surgical infections yet data are scanty or inconsistent [13].

The most important predicting factor of recurrence is the extent of surgical en-bloc resection: when simple excision is performed and hyoid bone remains intact, recurrences occur in 55.6% of the patients [35, 36]. As mentioned previously, recurrence rate of TGDC after Sistrunk procedure is around 16.4% (4.4–40.8%). Some authors suggested even the “extended Sistrunk procedure” [33]: this technique consists in the dissection en-bloc until the sternocleidomastoid muscle margin is encountered and proceeds until the pretracheal fascia and a 10-mm cuff of tongue base is removed in continuity with the specimen. However, due to the wider excision of underlying tissues, this procedure carries a higher risk of complications and damage to surrounding structures like recurrent laryngeal nerve, jugular vein and artery and the trachea [37]. Therefore, in order to perform a satisfactory and sufficient resection, the correct preoperative diagnosis of TGDC is crucial to be performed. Differential diagnosis include: cystic metastatic lymph node, dermoid cyst, second branchial cleft cyst or isthmic thyroid cyst [38]. US is the gold standard imaging to study TGDCs and it may help in the differential diagnosis with other neck masses; the presence of hyperechoic structures is typical of epidermoid cyst, even if TGDCs have not a decisive pattern. They may be heterogeneous (41.6%), anechoic (25%), homogenously hypoechoic (16.7%) or pseudosolid (16.7%) [39].

Eventually, histopathological analysis is an extremely useful tool in order to complete the diagnostic and therapeutic process. Indeed, the phenomenon of arborization has been reported by several studies [16, 40, 41], even though its role in recurrence is still controversial. The TGD can be located anteriorly to the hyoid bone, posteriorly to it or it can be enclosed within the bone itself. The anterior location appears to be the most common one, given the ventral pathway followed by the TGD during embryogenesis. TGD position is crucial, because the non-identification of a dominant duct during surgical removal may lead to persistence and recurrence. With respect to arborization, Chandra et al. suggested minor TGDs to be more susceptible to inflammation, leading to increased difficulty in surgical removal.

In our study, over 80% of patients presented arborization of the TGD at histopathological examination. In half of cases, the correct location of TGD with respect to the hyoid bone was not identified due to extensive inflammation. This finding confirms the aforementioned hypothesis of increased inflammation occurring along with arborization. The high proportion of arborization within the specimen might be related to the specific dissection carried out in between the cartilage portion of the hyoid, being its lateral parts not ossified when considering the pediatric population up to fifteen years of age [7]. On the other hand, only two patients presented recurrence of the disease: both of them presented arborization at histopathological analysis and had a history of recurrent inflammation.

## Conclusions

Surgery for TGDC remains a challenge for pediatric surgeons, while arborization was present in most of our cases which underwent surgery. With respect to the role of arborization, our study did not highlight sufficient conclusive data regarding their role in recurrence: instead, we report wide resection as satisfactory, being the arborization present in most of the cases at histopathology analysis. In order to minimize the risk of recurrence, other factors must be considered, the most crucial ones being: the degree of inflammation at surgery and history of inflammation.

## Data Availability

Data is available upon request from corresponding author.
